# Evaluation of Yahe^®^ and Panda^®^ 2.0 long-lasting insecticidal nets against wild pyrethroid-resistant *Anopheles gambiae s.l.* from Côte d’Ivoire: an experimental hut trial

**DOI:** 10.1186/s13071-021-04843-x

**Published:** 2021-07-01

**Authors:** Cyntia-Muriel Y. Clegban, Soromane Camara, A. Alphonsine Koffi, Ludovic P. Ahoua Alou, Jean-Paul Kabran Kouame, A. Fernand Koffi, Philippe K. Kouassi, Nicolas Moiroux, Cédric Pennetier

**Affiliations:** 1 Institut Pierre Richet/Institut National de Santé Publique (INSP), Bouaké, Côte d’Ivoire; 2 Université Félix Houphouët-Boigny, Abidjan, Côte d’Ivoire; 3 MIVEGEC, Univ Montpellier, CNRS, IRD, Montpellier, France

**Keywords:** *Anopheles gambiae s.l.*, Deltamethrin nets, Pyrethroid resistance, Côte d’Ivoire

## Abstract

**Background:**

Long-lasting insecticidal nets (LLINs) have played an important role in reducing the global malaria burden since 2000. They are a core prevention tool used widely by people at risk of malaria. The Vector Control Prequalification mechanism of the Word Health Organization (WHO-Vector Control PQ) established the testing and evaluation guidelines for LLINs before registration for public use. In the present study, two new brands of deltamethrin-impregnated nets (Yahe^®^ LN and Panda^®^ Net 2.0) were evaluated in an experimental hut against wild pyrethroid-resistant *Anopheles gambiae s.l.* in M’Bé nearby Bouaké, central Côte d’Ivoire.

**Methods:**

The performance of Yahe^®^ LN and Panda^®^ Net 2.0 was compared with that of PermaNet 2.0, conventionally treated nets (CTN), and untreated net to assess the blood-feeding inhibition, deterrence, induced exophily, and mortality.

**Results:**

Cone bioassay results showed that Panda^®^ Net 2.0, PermaNet 2.0 and Yahe^®^ LN (both unwashed and washed 20 times) induced > 95% knockdown or > 80% mortality of the susceptible *Anopheles gambiae* Kisumu strain. With the pyrethroid-resistant M’Bé strain, mortality rate for all treated nets did not exceed 70%. There was a significant reduction in entry and blood feeding (*p* < 0.05) and an increase in exophily and mortality rates (*p* < 0.05) with all treatments compared to untreated nets, except the CTNs. However, the personal protection induced by these treated nets decreased significantly after 20 washes. The performance of Panda^®^ Net 2.0 was equal to PermaNet^®^ 2.0 in terms of inhibiting blood feeding, but better than PermaNet^®^ 2.0 in terms of mortality.

**Conclusion:**

This study showed that Yahe^®^ LN and Panda^®^ Net 2.0 met the WHO Pesticide Evaluation Scheme (WHOPES) criteria to undergo phase III trial at the community level. Due to an increasing spread and development of pyrethroid resistance in malaria vectors, control of malaria transmission must evolve into an integrated vector management relying on a large variety of efficient control tools.

**Graphical Abstract:**

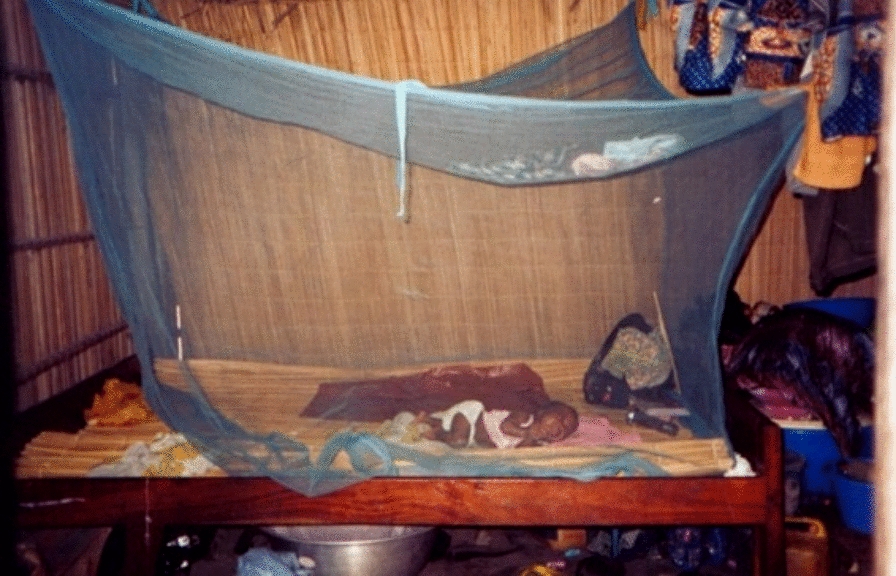

## Background

In the last two decades, malaria control efforts have yielded significant successes in many endemic countries by scaling up mass distribution of insecticide-treated mosquito nets (ITN) and large indoor residual spraying (IRS) campaigns to prevent malaria transmission, and providing access to effective artemisinin-based combination therapy (ACT) to treat malaria cases. These strategies have contributed substantially to global reduction in malaria morbidity and mortality [1]. Over 70% of this success was attributed to vector control, and ITNs on their own contributed 68% of the ~ 660,000 clinical malaria cases averted between 2000 and 2015 [2, 3].

Despite these gains, malaria transmission remains high, with an estimated 219 million cases and 435,000 deaths worldwide in 2019, of which 90% were reported in Sub-Saharan African countries. For this reason, the World Health Organization (WHO) has called for universal access to long-lasting insecticidal nets (LLINs) for all 3.2 billion people at risk of malaria worldwide [4, 5]. As a result, hundreds of millions of LLINs are distributed in malaria-endemic regions through various mechanisms every year. Currently, only pyrethroids are recommended for treatment of nets owing to their long residual activity, low cost, and safety [6, 7]. However, resistance to pyrethroids is spreading across Africa [8, 9] and has been reported in various localities of Côte d’Ivoire [10–12].

It must be stressed that, though resistance to pyrethroids is spreading across Africa [8, 9], and has been reported in various localities of Côte d’Ivoire [10–12], the effectiveness of LLINs in controlling malaria transmission at an operational level has not been called into question. However, the epidemiological outcomes of recent studies conducted in five countries showed that LLINs provided protection against malaria irrespective of resistance level [13, 14], supporting their continuous use in malaria-endemic areas in order to reduce the risk of infection [15].

Thus, LLINs remain the primary and best mass vector control tool for providing physical and chemical barriers to prevent malaria transmission in sub-Saharan Africa, when used properly and when in good condition [16]. The challenge is to maximize the benefits of the nets through improvement of their efficiency and durability, but also through the enhancement of population coverage (i.e. mass distributions every 3–4 years) and behavioural adherence (i.e. LLIN correct usage rate) [17].

Hence, the demand for high-quality LLINs has attracted the interest of several pesticide companies to produce new brands of LLINs. Two technological strategies are used in factories: insecticide can either be incorporated into the fibres or coated on the fibres [7]. Panda^®^ Net 2.0 and Yahe^®^ LN are two new brands of deltamethrin-treated LLINs developed by Life Ideas Biological Technology Co., Ltd.^©^ and Fujian Yamei Industry & Trade Co., Ltd.^©^, respectively. In Panda^®^ Net 2.0, deltamethrin is incorporated into 110 denier monofilament polyethylene fibres at a target dose of 1.8 g AI/kg, corresponding to 76 mg of deltamethrin per m^2^. In Yahe^®^ LN, deltamethrin is coated onto 75 denier monofilament polyester fibres at a target dose of 1.85 g AI/kg, corresponding to 55.5 mg of deltamethrin per m^2^.

Before any use in communities, new branded LLINs must go through an evaluation process and meet all efficacy and safety criteria before an interim or full approval is given by WHO-Vector Control PQ. Indeed, WHO guidelines state that LLINs must have effective insecticidal activity after 20 standard washes and a minimum lifespan of 3 years before a full or interim approval is given.

This paper reports on phase II experimental hut evaluations of Panda^®^ Net 2.0 and Yahe^®^ LN against wild free-flying *Anopheles gambiae s.l.* mosquitoes in M’Bé, nearby Bouaké (Central Côte d’Ivoire), an area of insecticide resistance.

The efficacy of the pyrethroid LLINs was assessed by evaluating the deterrence, induced exophily, blood-feeding inhibition, and mortality rates of wild *An. gambiae s.l.* entering the huts.

## Methods

### Study area and experimental hut design

The trial was carried out in M’Bé nearby Bouaké, an experimental station belonging to the Institute Pierre Richet. The M’Bé valley is a rice-growing area located 40 km north of Bouaké (5.209963 W and 7.970241 N) in the central region of Côte d’Ivoire. The mosquito population is composed of *An. gambiae s.l.*, *An. funestus*, *Culex* spp., and *Mansonia* spp. In the study area, *An. Coluzzi* is predominant among the *An. gambiae* complex. The *An. coluzzi* population displays resistance to organochlorides, pyrethroids, and carbamates, with an allelic frequency of the L104F *kdr* mutation of around 80% and the presence of metabolic resistance mechanisms [10, 11].

The experimental huts are made of concrete bricks, with a corrugated iron roof, a ceiling of thick polyethylene sheeting, and a concrete base surrounded by a water-filled moat to prevent entry of mosquito predators such as ants or spiders [18]. Mosquitoes, however, can readily enter through four window slits. These are made from pieces of metal, fixed at an angle to create a funnel with a 1-cm-wide gap. During each evaluation, the window slits are opened from 20:00 PM to 5:00 AM by the custodian. Mosquitoes fly upward to enter through the gap and then downward to exit the hut, thus impeding or greatly limiting the exit of the majority of mosquitoes that entered the hut. A single veranda trap made of polyethylene sheeting and screening mesh measuring 2 m long, 1.5 m wide, and 1.5 m high projects from the back wall of each hut. Movement of mosquitoes between the hut and veranda is unimpeded during the night.

### Net preparation and washing process

The two candidate LLINs, Panda^®^ Net 2.0 and Yahe^®^ LN, were supplied by Life Ideas Company Ltd.^©^ and Fujian Yamei Industry^©^, respectively. Washed and unwashed LLINs were evaluated using experimental huts to assess their effects on free-flying wild mosquitoes and for their ability to deter entry, repel or drive mosquitoes out of the huts, induce mortality, and inhibit blood feeding. WHO Pesticide Evaluation Scheme (WHOPES)-recommended LLINs, PermaNet^®^ 2.0 (which are coated with deltamethrin onto 75 denier monofilament polyester fibres at a target dose of 1.8 g AI/kg, corresponding to 55.5 mg/m^2^), were used as a positive control. An untreated net of 100 denier monofilament polyester fibres and nets conventionally treated (CTNs) with deltamethrin (25 mg AI/m^2^) washed to just before exhaustion were used as negative controls. The point of exhaustion was determined by washing CTNs using the phase II protocol [19]. The treated nets were washed at Institute Pierre Richet according to a protocol adapted from the standard WHO washing procedure used in phase I [19]. The interval of time required between two washes (i.e. regeneration time) was 3 days for Yahe^®^ LN and Panda^®^ Net 2.0 as established in phase I at the WHO collaborating centre in Montpellier. Nets were washed in aluminium bowls containing 10 L of well water and 2 g/L of soap ("savon de Marseille" like) using manual agitation. Each net was agitated for 6 min within a total washing/soaking period of 10 min. The net was agitated for 3 min, left to soak for 4 min and re-agitated for 3 min. Agitation was done by stirring the net with a pole at 20 rotations per minute. Rinsing was done twice using clean water (10 L per rinsing i.e. 20 L per net). Nets were dried horizontally in the shade then stored at ambient temperature between washes.

### Cone bioassays

Standard WHO cone bioassays were used to determine bio-efficacy of LLINs against a susceptible Kisumu and resistant M’Bé strain. A least three nets per treatment were bioassayed. The first round of bioassays were done on nets before washing. The second round of bioassays were conducted when all washes were completed, and a third one at the end of the hut experiments. Ten cones were placed on the five sections of each net (two per section). Five unfed mosquito females, 2–3 days old, were exposed for 3 min in each cone. Knockdown (KD) check was performed 60 min after exposure, and mortality was recorded 24 h after exposure. To determine the exhaustion point, the cone test was completed after each wash for the CTN until mortality and KD decreased below 80% and 95%, respectively.

### Experimental hut study design

The evaluation was run over 72 days between 4 August and 25 October 2014. The following comparison arms were tested:

- Untreated polyester net
- Unwashed Yahe^®^ LN
- Yahe^®^ LN washed 20 times
- Unwashed Panda^®^ Net 2.0
- Panda^®^ Net 2.0 washed 20 times
- Unwashed PermaNet^®^ 2.0
- PermaNet^®^ 2.0 washed 20 times
- CTN 1, polyethylene net with the same quality of fibres as that of Panda^®^ Net conventionally treated with deltamethrin and washed to just before exhaustion
- CTN 2, polyester net with the same quality of fibres as that of Yahe^®^ LN conventionally treated with deltamethrin and washed to just before exhaustion

Before testing in the experimental huts, six holes (4 cm × 4 cm) were made in each net (including control) to simulate the conditions of torn nets in the field: two holes in each of the long side panels and one hole at each end (head- and foot-side panels).

Adult volunteers entered the hut and slept under the nets from 8:00 PM to 6:00 AM six nights per week.

The treatment arms were rotated among the huts each week, and sleepers rotated each night according to a randomized Greco-Latin square scheme to minimize variations due to the hut and/or human attractiveness. At the end of the week, the huts were carefully cleaned and aired to avoid potential contamination. Each morning, resting and dead mosquitoes were collected from the inside of the nets, the room, and the veranda trap. The mosquitoes were morphologically identified at the species level using taxonomic keys [20]. The malaria vectors were scored by location as dead or alive and as fed or unfed. Live mosquitoes were placed in small cups for observation for 24 h with a sugar solution soaked in cotton wool.

Entomological parameters measured to assess the efficacy of the treatments in the experimental huts were:

- deterrence (i.e. the reduction in the number of mosquitoes collected in the huts with treated nets relative to the control huts);
- induced exophily (i.e. the reduction in the proportion of mosquitoes collected in the veranda traps relative to the control huts);
- blood-feeding inhibition (i.e. the reduction in the proportion of blood-fed mosquitoes in the huts with treated nets relative to the control huts);
- immediate and delayed mortality (i.e. the proportion of dead mosquitoes when collected in the morning and at 24 h after collection);
- personal protection (i.e. the reduction in the number of blood-fed malaria vectors collected in the treated arms relative to the negative control), calculated as follows:

% personal protection = 100 × (Bu − Bt/Bu),

where Bu is the total number blood-fed mosquitoes in the hut with untreated nets and Bt is the total number blood-fed mosquitoes in the hut with treated nets.

### Chemical analysis

At the end of the experimental hut trial, five pieces (25 cm × 25 cm) were cut from each net according to the WHO sampling method for LLINs and pooled for chemical analysis. All five net samples from each net were analysed separately to provide the average target concentration of the deltamethrin on each net. The analytical method used for determination of deltamethrin in samples was the CIPAC method 333/LN/(M2)/3 [21]. This method involves extraction of deltamethrin by refluxing for 30 min with xylene in presence of dibutyl phthalate as internal standard, solvent exchange to the mobile phase, and determination by high-performance liquid chromatography with UV diode array detection (HPLC–DAD).

### Statistical analysis

Mortality and KD rates from WHO cone bioassays were compared between each net using the Khi2 test. For statistical testing, the level of significance was set at 5%.

The proportional data from the hut trial (exophily, blood feeding, blood-fed and alive, and mortality) were analysed using generalized linear mixed models (GLMM) with the “brglm” function from the brglm package for R (version 3.3.2) using the bias-reduction method developed by Firth et al. [22]. The numbers of collected mosquitoes entering the huts (deterrence) and the numbers of blood-fed mosquitoes (personal protection) were analysed using a negative binomial mixed-effect model. Treatment arms were included as a fixed effect, and hut, sleepers, and weeks of collection were treated as random effects. Pairwise comparisons between the different treatment arms were performed using the “multcomp” package in R.

The non-inferiority of the Panda^®^ Net 2.0 and Yahe^®^ LN washed 20 times relative to PermaNet^®^ 2.0 unwashed and washed 20 times was tested according to WHOPES criteria [23], i.e. comparison of the 95% confidence intervals (CI) of the odds ratios (OR).

## Results

### Bio-efficacy of the treated nets (WHO cone bioassays)

**Fig. 1:**
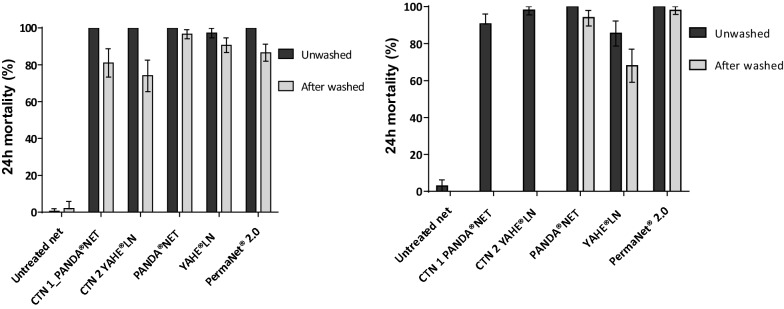
Mortality of insecticide-susceptible *An. gambiae s.l.* Kisumu strain after 3 min exposure to treated nets following WHO standard procedures (WHO 2013) run before (**a**) and after (**b**) the field trial. CTN 1 and CTN 2 were washed to just before exhaustion. Panda^®^ Net 2.0, Yahe^®^ LN, and PermaNet^®^ 2.0 were washed 20 times. Error bars represent 95% confidence intervals

**Fig. 2:**
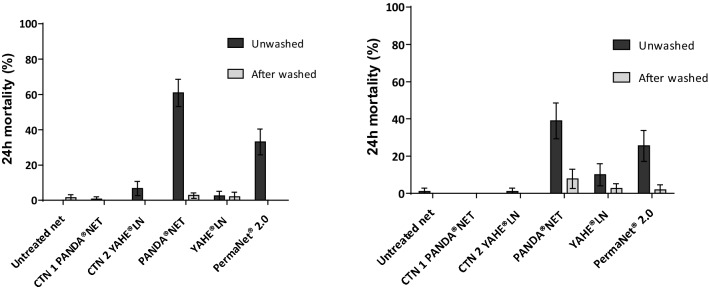
Mortality of wild insecticide-resistant *An. gambiae s.l.* M’Bé strain after 3 min exposure to treated nets following WHO standard procedures (WHO 2013) run before (**a**) and after (**b**) the field trial. CTN 1 and CTN 2 were washed to just before exhaustion. Panda^®^ Net 2.0, Yahe^®^ LN and PermaNet^®^ 2.0 were washed 20 times. Error bars represent 95% confidence intervals

Figures 1 and 2 present the proportions of susceptible and resistant mosquitoes dead 24 h after 3 min exposure to insecticide-treated netting in WHO cone bioassays.

Against the susceptible Kisumu strain, all treated nets were effective in terms of KD effect and mortality before any washing (Fig. 1). Mortality rates induced by Panda^®^ Net 2.0, PermaNet^®^2.0, CTN 1, and CTN 2 were all 100%, and 97.2% mortality was obtained by Yahe^®^ LN. After washing and before the field trial, KD decreased below the WHO threshold (95% KD) with all nets; however, mortalities were > 80% except CTN 2 (74%). Panda^®^ Net 2.0 and Yahe^®^ LN induced respectively 96.6% and 90.6% mortality, whereas the WHO-recommended PermaNet^®^2.0 induced 86.5% mortality (Fig. 1). After the field trial, mortality rates induced by all treated nets remained > 80% except for Yahe^®^ LN washed 20 times (68%).

When the pyrethroid-resistant M’Bé strain was used to evaluate LLINs, none of the nets reached the WHO criteria (knockdown rate ≥ 95% and mortality rate ≥ 80%). Mortality rates for all treated nets did not exceed 40% at any step, except Panda^®^ Net 2.0 which induced 60.9% mortality rate before washing and field trial. Mortality rates induced by Yahe^®^ LN unwashed and washed 20 times, Panda^®^ Net 2.0 and PermaNet^®^2.0 washed 20 times, and both CTNs were very low, ranging from 2 to 10% (Fig. 2).

### Experimental hut trial

During the 72 nights of collection during the trial, a total of 7621 wild free-flying female *An. gambiae s.l.* were collected in the experimental huts. The efficacy of all of the treatments with regard to deterrence, induced exophily, blood-feeding inhibition, and induced mortality is presented in Table 1.

**Table 1:** Summary results obtained against wild *An. gambiae s.l.* in experimental huts

	Untreated net	CTN 1	CTN 2	Panda^®^ Net 2.0 unwashed	Panda^®^ Net 2.0 washed 20X	PermaNet^®^ 2.0 unwashed	PermaNet^®^ 2.0 washed 20X	Yahe^®^ LN unwashed	Yahe^®^ LN washed 20X
Total females caught	1014	1098	1363	209	565	485	1388	478	1021
Average catch per night (95% CI)	11.0^d,e^ (7.0–17.5)	11.7^d,e^ (7.4–18.6)	15.4 ^e^ (9.8–24.3)	3.6^a^ (2.2–5.9)	7.3^b,c^ (4.5–11.7)	4.9^a,b^ (3.0–8.0)	13.8^d,e^ (8.7–21.7)	5.5^a,b^ (3.4–8.9)	9.8^c,d^ (6.1–15.5)
Deterrence (%)	–	−6.1	−39.7	67.8	33.8	55.5	−24.8	50.4	11.5
Total females in veranda	288	351	378	138	332	291	535	237	344
Exophily (%) (95% CI)	26.2 ^a^ (21.5–31.5)	30.6 ^a,b^ (25.4–36.2)	28.4 ^a,b^ (23.6–33.7)	64.0 ^e^ (55.3–71.9)	56.6^d,e^ (49.7–63.2)	59.1^e^ (52.0–65.8)	37.5 (31.9–43.4)	48.4^d^ (41.3–55.6)	34.2^b,c^ (28.6–40.3)
Induced exophily (%)	–	5.9	3.0	51.2	41.2	44.6	15.3	30.0	10.9
Blood feeding (%) (95% CI)	62.4^e^ (54.7–69.5)	64.2^e^ (56.7–71.1)	58.5^d,e^ (50.9–65.8)	19.7^a^ (13.6–27.7)	25.3^a^ (19.2–32.5)	25.9^a^ (19.7–33.4)	51.2^c^ (43.5–58.8)	39.5^b^ (31.5–48.0)	53.9^c,d^ (45.9–61.6)
Blood-feeding inhibition (%)	–	−3.0	6.2	68.4	59.5	58.4	17.9	36.7	13.6
Blood-fed and alive (%) (95% CI)	60.3^f^ (52.7–67.4)	62.5^f^ (55.1–69.4)	57.1^e,f^ (49.6–64.2)	9.5^a^ (5.9–15.0)	21.3^b^ (16.0–27.2)	22.0^b^ (16.5–28.8)	49.3^d^ (41.9–56.8)	38.0^c^ 30.3–46.3)	52.1^d,e^ (44.4–59.8)
Average female blood-fed per night (95% CI)	6.6^c,d^ (4.2–10.5)	7.3^c,d^ (4.6–11.7)	8.9^d^ (5.6–14.0)	0.8^a^ (0.4–1.4)	1.9^b^ (1.1–3.2)	1.4^a,b^ (0.8–2.3)	7.2^c,d^ (4.5–11.4)	2.2^b^ (1.3–3.6)	5.1^c^ (3.2–8.2)
Personal protection (%)	–	−10.8	−33.7	88.1	71.5	79.4	−8.6	67.3	22.5
Overall mortality (%) (95% CI)	8.5^a^ (6.4–11.2)	9.4^a,b^ (7.2–12.2)	8.4^a^ (6.4–10.9)	53.7^d^ (44.9–62.2)	25.3^c^ (20.2–31.2)	23.0^c^ (18.0–28.8)	9.8^a,b^ (7.6–12.6)	14.5^b^ (10.7–19.2)	10.6^a,b^ (8.1–13.9)
Corrected mortality (%)	–	1.0	−0.1	49.4	18.4	15.8	1.5	6.5	2.4

Untreated net (negative control) was used as reference category for the analysis. Values in the same row sharing the same letter superscript do not differ significantly at 5% level (GLM, *p* > 0.05)

Washed 20X: net washed 20 times

### Mosquito entry (deterrence) and exit (exophily) from huts

Compared to the untreated net, the number of female *An. gambiae s.l.* that entered the hut with treated nets was significantly reduced with four treated nets among the eight arms (Panda^®^ Net 2.0 unwashed, RR = 0.32 [CI 95 0.25–0.45], *p* < 0.0001; Panda^®^ Net 2.0 washed 20 times, RR = 0.66 [0.53–0.87], *p* = 0.0259; PermaNet^®^ 2.0 unwashed, RR = 0.44 [0.35–0.60], *p* < 0.0001; and Yahe^®^ LN unwashed, RR = 0.50 [0.39–0.67], *p* < 0.0001), indicating a significant deterrence effect against this malaria vector (Table 1). Nevertheless, four treated nets did not show any significant difference with the untreated net (CTN 1, RR = 1.06 [CI 95 0.86–1.36], *p* = 0.9999; CTN 2, RR = 1.40 [1.15–1.77], *p* = 0.0634; PermaNet^®^ 2.0 washed 20 times, RR = 1.25 [1.03–1.57], *p* = 0.5194; and Yahe^®^ LN washed 20 times, RR = 0.88 [0.72–1.14], *p* = 0.9802). When washed 20 times, PermaNet^®^2.0 and Yahe^®^ LN lost their deterrence effects compared to unwashed nets (PermaNet^®^ 2.0 washed 20 times, RR = 2.81 [2.24–3.73], *p* < 0.0001; and Yahe^®^ LN washed 20 times, RR = 1.79 [01.41–2.41], *p* = 0.0008).

The proportion of female *An. gambiae s.l.* exiting from huts with CTN 1 and CTN 2 did not differ significantly from that with untreated nets (CTN 1, OR = 1.24 [CI 95 1.01–1.51], *p* = 0.4549; CTN 2, OR = 1.12 [0.92–1.36], *p* = 0.9697) (Table 1), whereas there was a significantly higher induced exophily with Panda^®^ Net 2.0, PermaNet^®^ 2.0, and Yahe^®^ LN either washed or unwashed (OR between 1.46 and 5.01, *p* < 0.001) relative to an untreated net. When compared to unwashed nets, PermaNet^®^ 2.0 washed 20 times (OR = 0.41 [0.33–0.52], *p* < 0.0001) and Yahe^®^ LN washed 20 times (OR = 0.55 [0.44–0.70], *p* < 0.0001) induced significantly lower exophily. In contrast, the exophily rate of Panda^®^ Net 2.0 unwashed and washed 20 times did not differ significantly (OR = 0.73 [0.52–1.03], *p* = 0.6825).

### Blood-feeding inhibition and personal protection

The *An. gambiae s.l.* blood-feeding rates in the nine treatment arms is given in Table 1.

Blood-feeding rates with the untreated nets did not differ significantly from the CTN 1 (OR = 1.08 [0.90–1.31], *p* = 0.9957) or CTN 2 (OR = 0.85 [0.71–1.02], *p* = 0.7114). In contrast, all LLINs, both unwashed and washed 20 times, significantly decreased blood-feeding rates (0.15 < OR < 0.70, *p* < 0.0155). The lowest blood-feeding rate was recorded with the unwashed Panda^®^ Net 2.0 (Table 2). However, the blood-feeding inhibition did not differ significantly between the unwashed and washed Panda^®^ Net 2.0 (OR = 1.38 [0.93–2.04], *p* = 0.7937). The blood-feeding inhibition rates measured for PermaNet^®^ 2.0 and Yahe^®^ LN decreased significantly after washing 20 times (PermaNet^®^ 2.0 washed 20 times, OR = 2.99 [2.34–3.82], *p* < 0.0001; Yahe^®^ LN washed 20 times, OR = 3.33 [2.58–4.30], *p* < 0.0001). The 95% CI for the odds ratios showed that the blood-feeding rate with Panda^®^ Net 2.0 (both unwashed and washed 20 times) did not differ from that of the unwashed PermaNet^®^ 2.0, whereas both the unwashed Panda^®^ Net 2.0 and that washed 20 times induced higher blood-feeding inhibition than PermaNet^®^ 2.0 washed 20 times (Table 2).

**Table 2:** Results of non-inferiority statistical analysis of the performance of Panda^®^ Net and Yahe^®^ LN nets versus standard reference PermaNet^®^ 2.0

Outcomes	Treatment arms	PermaNet^®^ 2.0, unwashed Mean: 25.9 (19.7–33.4)	PermaNet^®^ 2.0, washed 20 times Mean: 51.2 (43.5–58.8)
Blood feeding	PermaNet^®^ 2.0, unwashed Mean: 25.9 (19.7–33.4)^a^	OR = 1	OR = 0.33 (0.26–**0.43**)
	PermaNet^®^ 2.0, washed 20 times Mean: 51.2 (43.5–58.8)^c^	OR = 2.99 (2.34–3.82)	OR = 1
	Panda^®^ Net 2.0, unwashed Mean: 19.7 (13.6–27.7)^a^	OR = 0.70 (0.47–**1.04**)	OR = 0.23 (0.16–**0.33**)
	Panda^®^ Net 2.0, washed 20 times Mean: 25.3 (19.2–32.5)^a^	OR = 0.97 (0.72–**1.30**)	OR = 0.32 (0.25–**0.41**)
	Yahe^®^ LN, unwashed Mean: 39.5 (31.5–48.0)^b^	OR = 1.86 (1.39–2.49)	OR = 0.62 (0.49–**0.79**)
	Yahe^®^ LN washed 20 times Mean: 53.9 (45.9–61.6)^c^	OR = 3.33 (2.58–4.30)	OR = 1.11 (0.93–**1.33**)
Total mortality	PermaNet^®^ 2.0, unwashed Mean: 23.0 (18.0–28.8)^b^	OR = 1	OR = 2.73 (**2.04**–3.67)
	PermaNet^®^ 2.0, washed 20 times Mean: 9.8 (7.6–12.6)^a^	OR = 0.37 (0.27–0.49)	OR = 1
	Panda^®^ Net 2.0, unwashed Mean: 53.7 (44.9–62.2)^c^	OR = 3.89 (**2.71–**5.58)	OR = 10.65 (**7.54**–15.04)
	Panda^®^ Net 2.0, washed 20 times Mean: 25.3 (20.2–31.2)^b^	OR = 1.14 (**0.85**–1.53)	OR = 3.11 (**2.37**–4.09)
	Yahe^®^ LN unwashed Mean: 14.5 (10.7–19.2)^a^	OR = 0.57 (0.40–0.80)	OR = 1.55 (**1.11**–2.17)
	Yahe^®^ LN, washed 20 times Mean: 10.6 (8.1–13.9)^a^	OR = 0.40 (0.30–0.54)	OR = 1.09 (**0.82**–1.45)

Bold values indicate non inferiority criterion

Non-inferiority of Panda^®^ Net 2.0 and Yahe^®^ LN combined feeding inhibition and 24 h mortality with PermaNet^®^ 2.0 as the reference. The candidate net is deemed non-inferior if (i) the upper 95% CI estimate of the odds ratio (OR) describing the difference in mosquito blood feeding between the candidate net and PermaNet 2.0 is greater than 1.43; (ii) the lower 95% CI estimate of the OR describing the difference in mosquito mortality between the candidate net and PermaNet 2.0 is greater than 0.7

Outcome values (mean) in the same column sharing the same letter superscript do not differ significantly at a 5% level (GLM, *p* > 0.05)

Personal protection relies on the number of blood-fed female *An. gambiae s.l.* collected in experimental huts with treated nets relative to experimental huts with untreated nets (Table 1). Personal protection rates given by unwashed LLINs were 88% for Panda^®^ Net 2.0, 79% for PermaNet^®^ 2.0, and 67% for Yahe^®^ LN. However, personal protection fell significantly after 20 washes to 72% for Panda^®^ Net 2.0, to 23% for Yahe^®^ LN, and to 0% for PermaNet^®^ 2.0.

### Mosquito mortality

Both CTN 1 (OR = 1.12 [0.82–1.52]) and CTN 2 (OR = 0.99 [0.73–1.34]) and both PermaNet^®^ 2.0 washed 20 times (OR = 1.17 [0.88–1.57]) and Yahe^®^ LN washed 20 times (OR = 1.28 [0.94–1.76]) failed to induce greater mortality relative to the untreated control net (GLM, *p* > 0.05, Table 1). In contrast, Panda^®^ Net 2.0 unwashed (OR = 12.50 [8.66–18.03], *p* < 0.0001) and washed 20 times (OR = 3.65 [2.70–4.95], *p* < 0.0001), PermaNet^®^ 2.0 unwashed (OR = 3.21 [2.34–4.42], *p* < 0.0001), and Yahe^®^ LN unwashed (OR = 1.82 [1.27–2.60], *p* = 0.0270) induced a substantial increase in mortality compared to the hut with the untreated control net, with mortality rates ranging from 15 to 54%. Panda^®^ Net 2.0 and PermaNet^®^ 2.0 LLINs washed 20 times induced less mortality than unwashed LLINs. (OR = 0.29 [0.21–0.42], *p* < 0.0001 for Panda^®^ Net 2.0 and OR = 0.37 [0.27–0.49], *p* < 0.0001 for PermaNet^®^ 2.0), whereas no significant difference was observed between the unwashed and washed Yahe^®^ LN. Nevertheless, the best killing effect was obtained with the unwashed Panda^®^ Net 2.0 (corrected mortality 49.4%), followed by Panda^®^ Net 2.0 washed 20 times (corrected mortality 18.4%). The unwashed Panda^®^ Net 2.0 performed better than the unwashed PermaNet^®^ 2.0 (OR = 3.89 [95% CI 2.71–5.58]; *p* < 0.001) (Table 2). It is interesting to note that Panda^®^ Net 2.0 washed 20 times was non-inferior to unwashed PermaNet^®^ 2.0 (OR = 0.97 [95% CI 0.72–1.30]; *p* < 0.001) (Table 2).

### Chemical analysis

The mean deltamethrin content in treated nets before washing, after washing, and after trialing in the huts is summarized in Table 3. The initial concentrations of deltamethrin in Panda^®^ Net 2.0, PermaNet^®^ 2.0 LN, and Yahe^®^ LN were close to the target dose of 1.8 g/kg ± 25% for Panda^®^ Net 2.0, 1.4 g/kg ± 25% for PermaNet^®^ 2.0 LN, and 1.85 g/kg ± 25% for Yahe^®^ LN with a variation of less than 10%, indicating a good homogeneity of the distribution of the active ingredient within the net. After washing, the deltamethrin content was 1.76 g AI/kg for Panda^®^ Net 2.0, 0.22 g AI/kg for PermaNet^®^ 2.0, and 1.39 g AI/kg for Yahe^®^ LN, corresponding to an overall wash retention rate of 79%, 15%, and 69% for Panda^®^ Net 2.0, PermaNet^®^ 2.0, and Yahe^®^ LN, respectively. After testing in the field, the deltamethrin content did not decrease significantly with either washed or unwashed nets.

**Table 3:** Results of chemical analysis of LLINs used in the experimental trial

LLIN	Deltamethrin content (g/kg)			
	Before washing	After washing	AI retention (% of wash 0)	After field trial
PermaNet^®^ 2.0, unwashed	–	–	–	1.32
PermaNet^®^ 2.0, washed 20 times	1.43	0.22	15	0.17
Panda^®^ Net 2.0, unwashed	–	–	–	2.14
Panda^®^ Net 2.0, washed 20 times	2.24	1.76	79	1.55
Yahe^®^ LN, unwashed	–	–	–	1.98
Yahe^®^ LN, washed 20 times	2.00	1.39	69	1.47
CTN 1 (at 25 mg/m^2^ exhausted)	1.06	0.02	–	0.02
CTN 2 (at 25 mg/m^2^ exhausted)	0.84	0.06	–	0.03

PermaNet^®^ 2.0 (deltamethrin coated onto polyester LN; 1.4 g/kg ± 25% [1.05–1.75 g/kg]), Panda^®^ Net 2.0 (deltamethrin incorporated into polyethylene LN; 1.8 g/kg ± 25% [1.35–2.25 g/kg]), Yahe^®^ LN (deltamethrin coated onto polyester LN; 1.85 g/kg ± 25% [1.39–2.31 g/kg])

### Perceived side effects

There were no reported negative side effects such as itching, dizziness, or nose running among the nine sleepers who participated in the experimental hut trial. The benefit perceived by them was undisturbed night sleep throughout the field trial due to the reduction of the inconvenience created by the presence of mosquitoes.

## Discussion

In a framework of a resistance management plan and cost-effectiveness of malaria control, the LLIN arsenal must include highly efficient LLINs impregnated with pyrethroid alone or in combination with either a non-pyrethroid compound or synergist compound such as piperonyl butoxide (PBO).

The current study was conducted in Bouaké, Côte d’Ivoire, in which a wild population of *An. gambiae s.l.* mosquitoes has been identified as having both target-site mutations and metabolic mechanisms conferring resistance to insecticides [11, 24, 25]. The study assessed, in field conditions, the efficacy of two brands of pyrethroid LLINs (Panda^®^ Net 2.0 and Yahe^®^ LN) impregnated with deltamethrin against wild pyrethroid-resistant *An. gambiae s.l.*

WHO cone bioassays conducted on susceptible *An. gambiae s.s.* Kisumu showed mortality rates > 80% with unwashed and washed Panda^®^ Net 2.0, Yahe^®^ LN, and PermaNet^®^2.0 before and after the hut trial. This indicates a great insecticidal efficacy of both Panda^®^ Net 2.0 and Yahe^®^ LN against susceptible *An. gambiae s.l.* mosquitoes and satisfied the WHO criteria (knockdown rate ≥ 95% and mortality rate ≥ 80%). In contrast, against pyrethroid-resistant M’Bé mosquitoes, none of the LLINs reached the WHO criteria. Low mortality induced by standard pyrethroid LLINs has already been reported in Côte d’Ivoire [26–29], in Burkina Faso [30], and Benin [31, 32] where the malaria vector *An. gambiae s.l.* displays high-level resistance to pyrethroids through a combination of target-site mutations (L1014F kdr, N1575 Y) [33] and metabolic mechanisms [10, 11, 32, 34].

Significant deterrence was evidenced for Panda^®^ Net (unwashed and washed 20 times), unwashed PermaNet^®^ 2.0, and unwashed Yahe^®^ LN. In contrast, CTN 1, CTN 2, PermaNet^®^ 2.0 washed 20 times, and Yahe^®^ LN washed 20 times did not display any effect on hut entrance. These results indicate that 20 washes decreased the deterrence effect for PermaNet^®^ 2.0 and Yahe^®^ LN, whereas 20 washes did not impact Panda^®^ Net’s deterrence. Nevertheless, we did not evidence any attractive effect among all treated bed nets as reported for some LLINs after washes [35]. The trend was different when we looked at the exophily. Indeed, all treated nets, either washed or unwashed, induced exophily, indicating that when a tarsal contact with the treated net is possible, the 20 washes did not impact on efficacy. This supports the increasing research interests in deciphering insecticide sensory detection in malaria vectors [36]. Blood-feeding rates allowed us to calculate personal protection conferred by each bed net. Panda^®^ Net (unwashed and washed 20 times), unwashed PermaNet^®^ 2.0, and unwashed Yahe^®^ LN did protect users against pyrethroid-resistant *An. gambiae s.l.* from M’Bé. It is worth noting that these nets were purposely holed to simulated torn nets, but they showed great protection despite the holes, even higher than reported by other studies conducted in the same area [26, 37]. These results suggests that in an area with highly resistant malaria vectors, pyrethroid LLINs still confer some personal protection [38].

In contrast, community protection mainly relying on the knockdown effect and mortality induced by LLINs is threatened by insecticide resistance mechanisms. Mortality of the host-seeking *An. gambiae s.l.* in experimental huts with all nets (except unwashed Panda^®^ Net) was < 20%. Unwashed Panda^®^ Net induced 49.4% and 18.4% mortality after 20 washes. This is better than other brands and CTNs, indicating that Panda^®^ Net 2.0 was non-inferior in terms of feeding inhibition and superior for mortality relative to PermaNet^®^ 2.0 as the reference product.

Though pyrethroid resistance is now widespread across Africa [39], LLINs are still the cornerstone of the fight against malaria transmission [2, 3]. A previous study conducted in M’Bé found that the alpha-cypermethrin-treated nets have conferred high personal protection against mosquito bites despite inducing low mortality [27]. In rural Tanzania, pyrethroid-treated nets did not kill moderately resistant *An. arabiensis* in experimental huts, but conferred high-level personal protection through simple bite prevention [40]. According to Okumu et al. [41], the barrier effects of LLINs and the sublethal effects of insecticides are sufficient to maintain LLIN effectiveness despite resistance. Nevertheless, insecticide resistance in malaria vectors has spread geographically and increased in intensity, particularly to pyrethroid insecticides [42]. In this context, there is a need for cost-effective technologies and new tools that can maintain the efficacy of currently available tools or complement them in order to better fight resistant malaria vectors. Next-generation LLINs treated with a mixture of pyrethroid and PBO or a non-pyrethroid insecticide are being developed or evaluated. These LLINs are designed to overcome pyrethroid resistance and will be of great utility in a broader resistance management strategy. Some of these LLINs are now available as PermaNet^®^ 3.0, Olyset^®^ Plus, Olyset^®^ Duo, Interceptor^®^ G2, or Veeralin^®^) and have shown their efficacy even in known strong insecticide-resistant areas like Côte d’Ivoire [28, 29, 43], Benin [44, 45], and Burkina Faso [46]. Resistance management could also rely on combination of intervention in order to target surviving malaria vectors with all ecological and behavioural diversity. Such a general insecticide resistance management plan must be dynamic, using a wide range of vector control tools in effective combination on the basis of resistance monitoring data [47].

## Conclusion

In the current study, Panda^®^ Net 2.0 and Yahe^®^ LN fulfilled the WHO-PQ criteria for phase II studies of LLINs. These two nets were pre-qualified by WHO, like PermaNet^®^ 2.0, which confers effective personal protection against mosquito bites despite its low killing effect in areas with high pyrethroid resistance in malaria vectors. They are now integrated into the vector control tool arsenal available for an integrated vector management.

## Data Availability

All data generated or analysed during this study are included in this article.
